# The influence of microbial physiology on biocatalyst activity and efficiency in the terminal hydroxylation of *n*-octane using *Escherichia coli* expressing the alkane hydroxylase, CYP153A6

**DOI:** 10.1186/1475-2859-12-8

**Published:** 2013-01-25

**Authors:** Oluwafemi A Olaofe, Caryn J Fenner, Rama Krishna Gudiminchi, Martha S Smit, Susan TL Harrison

**Affiliations:** 1 Centre for Bioprocess Engineering Research (CeBER), Department of Chemical Engineering, University of Cape Town, Private Bag X3, Rondebosch 7701, Cape Town, South Africa; 2 Department of Microbial, Biochemical and Food Biotechnology, University of the Free State, Bloemfontein, South Africa; 3 South African DST-NRF Centre of Excellence in Catalysis, c*change, University of Cape Town, Private Bag, Rondebosch 7701, Cape Town, South Africa

**Keywords:** Octane, 1-Octanol, CYP153A6, Whole cell biocatalysis, Alkane hydroxylation

## Abstract

**Background:**

Biocatalyst improvement through molecular and recombinant means should be complemented with efficient process design to facilitate process feasibility and improve process economics. This study focused on understanding the bioprocess limitations to identify factors that impact the expression of the terminal hydroxylase CYP153A6 and also influence the biocatalytic transformation of *n*–octane to 1-octanol using resting whole cells of recombinant *E*. *coli* expressing the CYP153A6 operon which includes the ferredoxin (Fdx) and the ferredoxin reductase (FdR).

**Results:**

Specific hydroxylation activity decreased with increasing protein expression showing that the concentration of active biocatalyst is not the sole determinant of optimum process efficiency. Process physiological conditions including the medium composition, temperature, glucose metabolism and product toxicity were investigated. A fed-batch system with intermittent glucose feeding was necessary to ease overflow metabolism and improve process efficiency while the introduction of a product sink (BEHP) was required to alleviate octanol toxicity. Resting cells cultivated on complex LB and glucose-based defined medium with similar CYP level (0.20 μmol g_DCW_^-1^) showed different biocatalyst activity and efficiency in the hydroxylation of octane over a period of 120 h. This was influenced by differing glucose uptake rate which is directly coupled to cofactor regeneration and cell energy in whole cell biocatalysis. The maximum activity and biocatalyst efficiency achieved presents a significant improvement in the use of CYP153A6 for alkane activation. This biocatalyst system shows potential to improve productivity if substrate transfer limitation across the cell membrane and enzyme stability can be addressed especially at higher temperature.

**Conclusion:**

This study emphasises that the overall process efficiency is primarily dependent on the interaction between the whole cell biocatalyst and bioprocess conditions.

## Background

**Figure 1:**
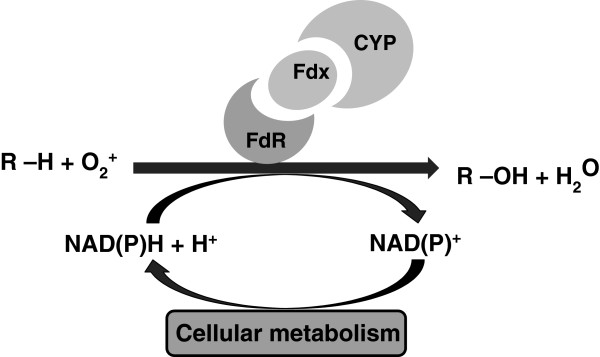
**The schematic representation of the various reactions of the alkane hydroxylase protein (CYP153A6) and its electron transfer system within a whole cell. **The ferrodoxin reductase (FdR) transfers electrons from NAD(P)H through a small electron transfer protein, ferrodoxin (FdX), to the hydroxylase protein CYP153A6 for the oxidation of the alkane substrate to its alcohol equivalent.

The linear paraffins from expanding activities in the petrochemical industry provide an inexpensive hydrocarbon feedstock. The use of micro-organisms as biocatalysts for the production of value added chemicals using this non-conventional substrate has gained interest due to the difficulty in achieving efficient process economics with chemical catalysts owing to the inert nature of the reactant. Cytochrome P450 monooxygenases (CYPs) have the ability to hydroxylate a wide range of substrates by the regio-and/or stereo-specific insertion of an oxygen atom into a carbon-hydrogen bond at physiological pressure and temperature (Figure 1). This leads to reduced by-product and waste generation, and thereby potentially reduced environmental burden. The use of this enzyme as biocatalyst on a large scale is hampered by factors such as low enzyme activity, substrate and product toxicity, biocatalyst stability and the requirement for reduced co-factors [1-3]. Various studies have been conducted to address the aforementioned shortcomings using whole cells, the preferred option over free enzymes for alkane activation. These have used a selection of enzymes from the CYP family. The CYP153s have the advantage of terminal alkane hydroxylation with selectivity of over 95%, a notable advantage over biocatalysts performing similar functions including the well-studied alkane mono-oxygenase (alkB) from *Pseudomonas*. They catalyse the hydroxylation of lower to medium chain linear alkanes, C_4_-C_11_, with octane as preferred substrate [4-8].

In biocatalysis, productivity is a function of biocatalyst activity. This can be maximised by protein engineering using molecular techniques or by adopting a bioprocess-based approach. To date, most studies on CYPs as biocatalysts have focused on the use of molecular methods to manipulate and improve enzyme properties, with the process and reaction engineering given less attention. Molecular methods for enzyme engineering such as rational design, directed evolution and site directed mutagenesis have been reported to yield up to 8-fold improvement for CYP whole-cell processes while increments of over 25-fold have been achieved by considering factors such as microbial host selection, reaction and process optimisation [3,9-15]. For the hydroxylation of alkanes using whole cell biocatalysts expressing CYP153s, significant studies have centred on the choice of host organism, vector systems and other molecular tools to improve protein expression and whole cell activity [4,5,8,16-18]. Although this has led to meaningful progress in biocatalyst design, productivity in most *in vivo* alkane biotransformation studies fall outside the operating window described as essential for fine chemical synthesis, a minimum of 0.1 g^-1^ l^-1^ h^-1^[12]. The system is often characterised by poor activity and low stabilities, resulting in short reaction time and unacceptable productivity. In a recent study to improve the recombinant expression of CYP153A6 using a pET vector, Gudiminchi *et al*. [19] reported notable improvements in biocatalyst activity and productivity. In this study, the major influence resulted from biocatalyst loading, increase in oxygen content and choice of carbon source to supply energy and reducing equivalents for biotransformation. Although the biocatalyst efficiency achieved was over 12 fold better than literature values, the factors constraining further process improvement were not well understood. From the studies, it was apparent that the overall process efficiency is dictated by the physiological state of the cell, determined by the interaction between whole cell biocatalyst, bioprocess conditions and their history. The physiology of whole cell biocatalyst during long term biotransformation is largely unknown and may also be responsible for dynamic biocatalyst activities and stabilities, decreasing predictability of the bioprocess. These physiological factors include overexpression of the cloned gene(s), supply of nutrients and growth factors through the growth medium and mass transfer, product toxicity, and cofactor regeneration [20,21]. These environmental process conditions determine the extent to which the maximum active enzyme concentration achieved in cell cultivation is exploited in the alkane bioconversion phase. Therefore, in conjunction with strain development and metabolic engineering, it is important that parameters that constrain physiological state and subsequent process performance be considered to improve the specific biocatalyst activity and productivity. These include cell environmental factors that impact the expression level of the cloned gene, biocatalyst activity and stability.

In this study, the expression of the CYP153A6 gene product in *E*. *coli*, its activity, stability and octane biotransformation to 1-octanol were investigated as a function of induction conditions, FeCl_3_ concentration, temperature, glucose availability to facilitate co-factor regeneration and product toxicity. The influence of overflow metabolism and product toxicity were assessed to inform further process development.

## Results

### Expression of CYP153A6 in a batch process

#### Temperature

For the growth temperature studies, a flask culture was allowed to proceed at the same temperature for both the growth and expression phases while a second flask was incubated at the test temperature for growth and a reduced temperature of 20°C after induction for enzyme expression. The results presented in Table 1 show that there was no substantial difference in CYP153A6 concentration (0.16 to 0.20 μmol per g dry cell weight, DCW) when pre-induction temperatures of 20, 25, 30 and 37°C were used, giving a final octanol concentration of 0.80 – 1.00 g g_DCW_^-1^. When incubation following induction was continued at the initial growth temperature, CYP450 expression was affected negatively at the higher temperatures (25 – 37°C). The concentration of active enzyme in the cells incubated at 25°C was reduced by 3-fold relative to that at 20°C, leading to a 50% decrease in 1-octanol formation. No active P450 enzyme was observed in the cells incubated at 30 and 37°C. This was confirmed at the bioconversion stage with no octanol detected in biotransformations with cells following CYP expression at 30 and 37°C following induction.

**Table 1:** **Effect of temperature conditions on growth rate**, **CYP153A6 expression and subsequent bioconversion of octane to 1**-**octanol in a 72 h reaction**

**Temperature**	**Maximum growth rate**	**[CYP]**		**1-Octanol**	
**(°C)**	**μ**_**max**_**(h**^**-1**^**)**	**(μmol g**_**DCW**_^**-1**^**)**		**(g g**_**DCW**_^**-1**^**)**	
		**A**	**B**	**A**	**B**
20	0.20	0.17	0.16	1.00	1.00
25	0.37	0.20	0.06	1.00	0.50
30	0.50	0.17	0.00	0.80	0.00
37	0.82	0.16	0.00	0.85	0.00

Under condition A, the temperature was maintained as specified prior to induction, whereafter incubation was at 20°C. Under condition B, the specified temperature was maintained throughout cultivation, expression and bioconversion. DCW, dry cell weight.

#### Induction conditions in a batch process

**Figure 2:**
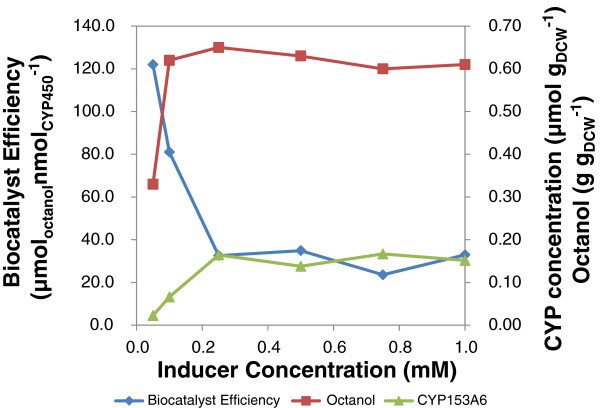
**Effect of IPTG and δ-ALA concentration on CYP153A6 expression, whole**-**cell octane biotransformation in 70 h and the resultant biocatalyst efficiency with cells grown on defined medium.** Dry biomass concentration was an average of 4.5 ± 0.2 g l^-1 ^across inducer concentrations.

The mid exponential phase of cell growth was determined as the preferred point for addition of inducer for optimal expression and activity of CYP153A6 by LB-grown cultures (data not shown). Further, the minimum effective inducer (IPTG) and heme precursor (δ-ALA) concentrations per unit biomass were determined by simultaneously varying IPTG and δ-ALA concentrations from 0.05 to 1 mM (10 to 220 μmol g_DCW_^-1^). A defined medium containing glucose, adapted from Pflug *et al*. [14] was selected for this study to improve the volumetric CYP153A6 concentration through increasing the biomass concentration. The P450 content, 1-octanol over a biotransformation period of 70 h and biocatalyst efficiency are presented in Figure 2. Relative to the control run (no induction), cell induction at mid-exponential phase did not inhibit biomass formation (an average of 4.5 ± 0.2 g l^-1^) across the range of inducer concentration studied (0.05 to 1 mM). The maximum CYP153A6 expression of 0.80 μM, translating into 0.20 μmol g_DCW_^-1^ was observed in the inducer concentration range of 0.25 to 1.00 mM. In biotransformations, the maximum biocatalyst efficiency (octanol formed per unit of active CYP153A6) of 120 μmol nmol^-1^ was observed when the IPTG concentration was 0.05 mM. This dropped with increasing CYP concentration, remaining constant above an IPTG concentration of 0.25 mM as the CYP concentration remained constant. The reduction in specific product formation with increased CYP153A6 expression at higher inducer concentration suggests a limiting factor in the octane bioconversion process.

**Figure 3:**
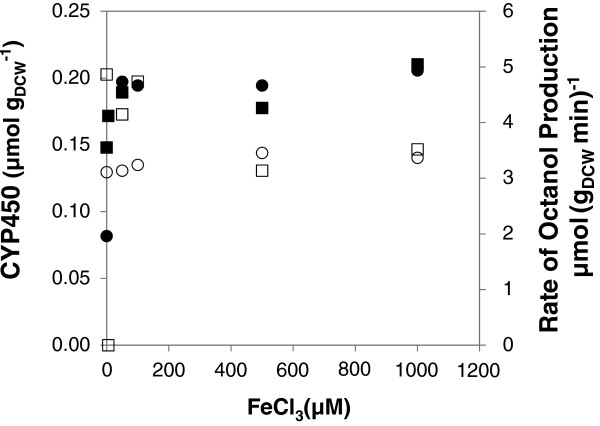
**Impact of FeCl**_**3 **_**concentration on CYP153A6 expression in LB {**■**} and glucose based defined medium {**●**}.** Harvested biomass was re-suspended in 0.2 M Sodium Phosphate buffer containing glucose and used for octane biotransformation. Rate of octanol production was determined using biomass from both LB {□} and chemically defined medium {○} at 23°C.

The impact of iron on the expression of the hemoprotein, CYP153A6, and whole cell biocatalyst activity was investigated by varying the FeCl_3_.6H_2_O concentration from 0 to 1000 μM. FeCl_3_.6H_2_O was added with IPTG and δ-ALA at the time of induction in both LB and glucose-based defined medium. The data presented in Figure 2 show that FeCl_3_ addition caused more than a 2-fold increase in CYP concentration in the defined medium, even at the minimum concentration of 50 μM. Further increase in FeCl_3_ concentration up to 1000 μM did not improve CYP concentration which remained at around 0.20 μmol g_DCW_^-1^. This trend was not reflected in the hydroxylation of octane by the resting cells as whole cell activity was in the range of 3 to 3.5 μmol_octanol_ (g_DCW_ min)^-1^, with or without FeCl_3_ addition (Figure 3).

The results from LB complex medium did not show a major difference in CYP expression (0.17 to 0.19 μmol g_DCW_^-1^) with FeCl_3_ concentration in the range of 5 to 1000 μM relative to the control without FeCl_3_ added (0.15 μmol g_DCW_^-1^). The rate of octanol formation varied between 4 and 5 μmol (g_DCW_ min)^-1^ with FeCl_3_ in the range of 0 to 100 μM. A further increase in iron concentration resulted in reduced biocatalyst activity (Figure 3).

#### Whole cell hydroxylation of n-octane

Further experiments to understand limiting factors in octane bio-oxidation were conducted using resting cells grown in LB and glucose-based chemically defined medium. The conditions that favour the maximum expression of CYP153A6 were also adopted for cell cultivation and protein expression prior to the use of biomass in bioconversion.

#### Temperature - rate of reaction and biocatalyst stability

To investigate the effect of temperature on the bioconversion of octane using resting *E*. *coli* cells, the bioprocess temperature was varied from 20 to 37°C. The maximum rate of product formation and the thermal stability of the whole cell biocatalyst were determined. The maximum biocatalyst activity of 16 μmol_octanol_ (g_DCW_ min)^-1^ was achieved at 37°C, with the octanol concentration reaching 0.48 g g_DCW_^-1^ in 6 h (Table 2). This represents a 4 fold increase over the value achieved at 20°C and previously reported by Gudiminchi *et al*. (2012). However, octanol accumulation was limited by biocatalyst instability at the higher temperature. Attempts to alleviate protein instability through the addition of glycerol did not result in any positive influence.

**Table 2:** **Comparison of key bioprocess parameters obtained in this study with previous reports on 1**-**octanol production using *E***. ***coli *and cytochrome P450**, **especially the CYP153 operon**

	**Fujii *et al., *2006**	**Baik, 2010**	**Bordeaux *et al., *2011**	**Koch, et al., 2009**	**Gudiminchi, 2012**	**This study**^**a**^	**This study**	**This study**^**b**^
*E*. *coli *biocatalyst	CYP153Aci	CYP, Fdx& Fdr	CYP153A13	CYP153A6	CYP153A6	CYP153A6	CYP153A6	CYP153A6
Growth medium	L-Broth	Marine Broth	n/a	Semi-defined	LB	LB	LB	Defined medium
Bioreactor	Vial	Shaker	n.a.	Vial	Vial	Vial	Vial	Vial
Temperature (°C)	28	37	23 ± 1	25	20	20	37	20
Biomass concentration	n.a.	n.a	n.a.	3.5	11	5.4	7.4	4.1
(g L_BRM_^-1^)								
Max octanol formed	2.3	0.45	n.a.	0.01	8.8	6.5	2.2	6.0
(g L_BRM_^-1^)								
Time max octanol (h)	24	6	2	1	24	48	6	120
Max volumetric rate	0.1	0.07	n.a.	0.01	0.32	0.16	0.82	0.08
(g L_BRM_^-1^ h^-1^)								
Biocat efficiency at max octanol_l_	n.a.	n.a.	0.06	0.003	0.80	1.20	0.46	1.45
(g_octanol_ g_DCW_^-1^)								
Max specific octanol formation rate per biomass	n.a.	n.a.	3.9	0.37	4.0	4.2	16	2.40
(μmol min^-1^ g_DCW_^-1^)								
Period_max rate_ (h)	0 – 24	2 – 6	0 – 2	0 – 1	0 – 24	0 – 24	0 – 4	24 – 48

^(a)^Figure 5A ^(b)^Figure 6.

#### Glucose fed-batch process

**Figure 4:**
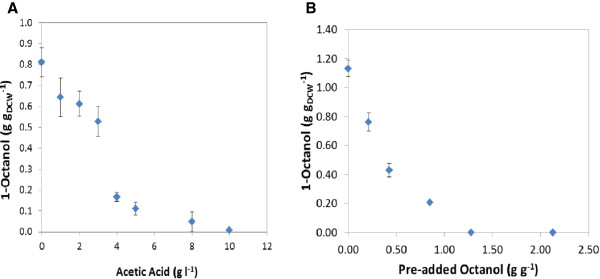
**Whole cell biocatalyst inhibition studies conducted by pre**-**adding (A) acetic acid (B) 1**-**octanol,to the reaction mixture at the start of octane hydroxylation. **Biomass concentration was 4 g L_BRM_^-1 ^with an initial glucose concentration of 5 g l^-1^. The end-point concentration was determined after 75 h of reaction in 40 ml amber vials.

**Figure 5:**
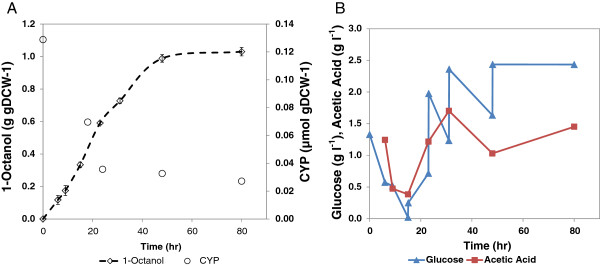
**(A) Whole cell biotransformation of octane with intermittent glucose feeding using a biomass concentration of 3.6 g L**_**BRM**_^**-1 **^**in a 40 ml vial using 1 ml bioreaction mixture and 300** **μl octane. **Active CYP153A6 was also observed over duration of reaction. **(B) **Glucose concentration and acetic acid formation were monitored.

The effective utilisation of glucose as energy source for product formation is necessary for process efficiency of bioconversion [22]. The preferred use of glucose as energy source by this whole cell biocatalyst was highlighted in a previous study [19]. It was, however, also demonstrated that increasing the glucose concentration beyond a critical value did not result in improved octanol accumulation, suggesting over-metabolism towards by-product formation. It was observed in several experiments that acetic acid (an indicator of overflow metabolism) reached a concentration of up to 2.5 g l^-1^ when glucose was added above a concentration of 10 g l^-1^ (1.0 g g_DCW_^-1^). It lowered the pH of the reaction mixtures from pH 7.2 to 5.6. This concentration of acetic acid was confirmed as partially inhibitory (by 35%) towards octanol formation in an experiment in which different concentrations of acetic acid (up to 10 g l^-1^) were added to the reaction mixtures (Figure 4A). Further investigation was conducted to ease overflow metabolism and also alleviate “glucose uncoupling” from the desired product formation by adopting a fed-batch approach. In this experiment, a glucose concentration in the range of 2 to 5 g l^-1^ (0.5 to 1.0 g g_DCW_^-1^) was maintained through intermittent glucose supplementation in fed- batch to prevent limitation (Figure 5B). In Figure 5A, it is seen that octanol accumulation of 0.9 g g_DCW_^-1^ was achieved in 48 h at a maximum rate of 3.17 μmol (g_DCW_ min)^-1^. Acetic acid formation was also reduced by 40% to 1.5 ± 0.2 g l^-1^. Although this result implies that this by-product is not the only factor limiting increased octanol accumulation, the process efficiency was improved by reducing over-oxidation of glucose to by-products.

#### Product toxicity using cells grown on LB and glucose based defined medium

**Figure 6:**
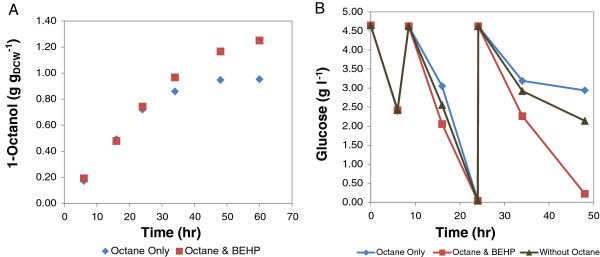
**(A) Whole cell hydroxylation of *n–*octane using biomass from LB cultures with *in situ *product removal using bis (2-ethylhexyl) phthalate. **The bio-reaction mixture consisted of 1 ml re-suspended cells (5 g_DCW _l^-1^) in 0.2 M sodium phosphate buffer, pH 7.2, and 30% v/v organic phase consisting of 100 μl BEHP. **(B) **Glucose concentration was monitored in each of the systems (with and without co-solvent) as well as a control system (without organic phase). The vials were opened intermittently for glucose addition and also to allow air inlet to prevent oxygen limitation. The reaction was mixed on an orbital shaker at 200 rpm and 20°C.

To understand the impact of product toxicity on octanol accumulation, 1-octanol was pre-added to the reaction mixture in the concentration range of 0 to 50 mM (0 to 2 g g_DCW_^-1^) and the reaction was allowed to continue for 72 h using cells grown in LB medium. The results show that product toxicity was experienced with increasing octanol concentration leaving about 17% product formation capacity at octanol concentration of 0.8 g g_DCW_^-1^ and no conversion at 1.25 g g_DCW_^-1^ (Figure 4B). Although externally added product may not mimic the effects of intracellularly produced octanol completely, the results confirm the inhibitory nature of the product. To alleviate this effect, a second organic phase was investigated for *in situ* product recovery. Two solvents, 1-hexadecene and bis (2-ethylhexyl) phthalate (BEHP) were used for this study. BEHP was preferred as a carrier solvent owing to its ability to maintain the maximum conversion rate and to the apparent alkane mass transfer limitation experienced with hexadecene. The maximum 1-octanol produced with hexadecane was limited to only 0.30 g g_DCW_^-1^ in 60 h. With BEHP as a carrier solvent, the maximum rate of product formation was maintained at 4 μmol_octanol_ (min g_DCW_)^-1^ reaching a concentration of 1.20 g g_DCW_^-1^ in 48 h. This represents a 50% increase in overall conversion over that achieved without a carrier solvent under the same condition (Figure 6A, Table 2). The product toxicity was less intense when BEHP was added as carrier solvent as depicted by the cell metabolism illustrated by glucose utilisation (Figure 6B). The rates of glucose consumption for the resting whole cell biocatalyst in glucose solution without substrate, and the resting whole cells in glucose solution with octane or octane and BEHP added were similar up to 24 h (0.48 mmol g_DCW_^-1^ h^-1^). However, a difference in glucose utilisation was noticed in the second 24 h period, especially between 34 to 48 h. During this period the glucose consumption rate was the highest for the resting cells with octane and BEHP added. These cells consumed almost twice as much glucose as the control cells without substrate, demonstrating that the whole cell biocatalyst was able to increase its metabolism to respond to increased carbon demand for the increased cofactor regeneration required by octane hydroxylation. The glucose consumption rate of the cells with only octane added was the lowest, showing that the resting whole cell biocatalyst was susceptible to product inhibition if a product sink was not available to reduce its concentration in the aqueous phase. However, octanol accumulation levelled off after 48 h apparently due to the decrease in pH from 7.2 to 5.4 ± 0.2.

**Figure 7:**
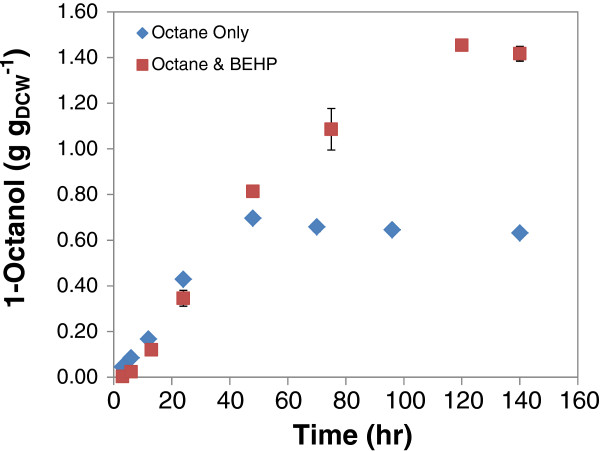
**Whole cell hydroxylation of *n***-**octane using resting cells grown on glucose based defined medium.** The biomass was harvested and re-suspended (4 g_DCW_ l^-1^) in 0.2 M Sodium Phosphate buffer, pH 7.2 consisting of glucose (starting concentration of 5 g l^-1^). A 1 ml aliquot of this mixture was introduced into the 60 ml amber vials and 30% v/v organic phase consisting of 200 μl octane and 100 μl BEHP was added. In another set, only octane (300 μl) was added. The vials were opened intermittently for glucose addition and also to allow inlet of air to prevent oxygen limitation. The reaction was mixed on an orbital shaker at 200 rpm and 20°C.

A similar experiment was carried out with cells cultured on defined medium. This experiment again demonstrated the effect of product inhibition clearly and its reduction by product removal into the BEHP phase. When no BEHP was added, product formation was almost linear for more than 25 h but ceased after 48 h. In comparison, it remained almost linear in excess of 50 h when BEHP was added and continued to be formed until 120 h. Thus without a carrier solvent, a maximum octanol concentration of 0.65 g g_DCW_^-1^ was achieved after 60 h, compared with 1.10 g g_DCW_^-1^ at 60 h and a maximum octanol concentration of 1.45 g g_DCW_^-1^ after 120 h when BEHP was added as co-solvent (Figure 7, Table 2).

**Figure 8:**
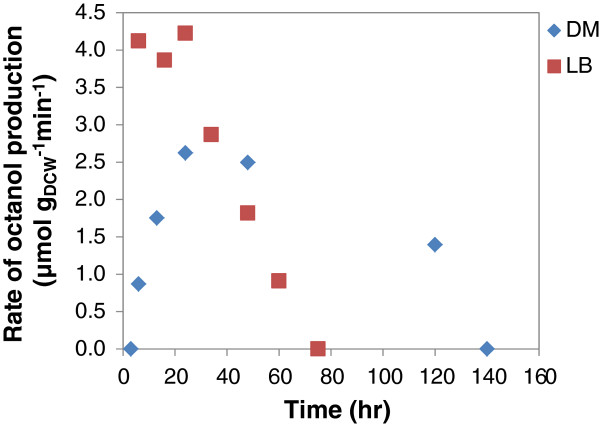
**Comparison of rate of octanol formation between cells cultivated on LB and defined medium**, **and subsequently used for octane biotransformation in 60 ml amber vials.**

Notable features of the reactions carried out with cells grown in chemically defined medium were the slower reaction rate and the longer time for which the biocatalyst remained active when compared with the LB grown cells. The result presented in Figure 8 shows that maximum activity was maintained at its highest value for the first 24 h with LB cells, whereafter it decreased linearly, exhibiting no activity at 75 h. For cells cultured on defined medium, the whole cell biocatalyst activity gradually increased, peaking at approximately 2.5 μmol_octanol_ (min g_DCW_)^-1^ at 24 h where it was maintained until 48 h before it decreased gradually to 1.5 μmol_octanol_ (min g_DCW_)^-1^ at 120 h, and rapidly decreased to a nil point at 140 h. Since the CYP concentration was initially the same in both LB and defined medium (0.20 μmol g_DCW_^-1^), this result suggests that the different medium composition conferred a different physiological state on the cells, thereby affecting their metabolic response and hydroxylation capability in octane biotransformation.

#### Cell permeabilisation and biocatalyst activity

The potential limiting effect of alkane transfer into the cells was studied by permeabilising the cell membrane using acetone or toluene in the concentration range of 5 to 20% v/v. Reactions were conducted at 23 and 37°C. Initial results with toluene or high concentrations of acetone (>5% v/v) showed cell aggregation and whole cell inactivation. No octanol formation was observed (data not shown). Further studies were conducted with 5% acetone. The results presented in Table 3 show the influence of acetone pre-treatment on the biotransformation rate. At 23°C, cells grown in either LB or chemically defined medium showed a two fold increase in octanol formation rate with acetone pre-treatment. This trend was also observed with defined medium grown cells at 37°C. On LB at 37°C, a decrease in whole cell biocatalytic activity was apparent. This suggests that acetone increases the permeability of the cell membrane towards the alkane substrate, thereby increasing mass transfer rates. The difference observed with LB grown cells at higher temperature suggests some physiological limitation which is explained later.

**Table 3:** **Hydroxylation of *n***-**octane using permeabilised cell in 60 ml amber vials**

**Medium**	**Defined**	**Defined**	**Defined**	**Defined**	**LB**	**LB**	**LB**	**LB**
**Pre**-**treatment**	None	Acetone	None	Acetone	None	Acetone	None	Acetone
**Temperature (°C)**	23	23	37	37	23	23	37	37
**Max specific octanol formation rate per biomass**	4.17	8.47	6.59	11.11	5.26	9.10	16.09	14.97
**(μmol min**^**-1**^**g**_**DCW**_^**-1**^**)**								

NB: Cell concentration, 3 – 4 g_DCW _l^-1^; reaction time, 2 h at 37°C & 6 h at 23°C; initial CYP concentration, 0.20 μmol g_DCW_^-1^.

The cells were pre-treated with acetone (5% v,v) prior to biotransformation.

## Discussion

### Expression of CYP153A6 in recombinant *E*. *coli*

Studies were carried out to inform the bioprocess factors that govern the biocatalytic activity of CYP153A6 in recombinant *E*. *coli* and its effect on the accumulation of 1-octanol in the biotransformation of *n*-octane and manipulation of these factors to improve biocatalyst activity and efficiency. The role of cultivation parameters such as temperature, induction conditions and supply of nutrients were investigated with respect to its effect on CYP153A6 expression driven by the T7 promoter in a recombinant *E*. *coli* BL21(DE3) strain carrying the pET28b-PFR1500 plasmid. Performance of the resultant whole cell biocatalyst under various biotransformation conditions was determined.

Reduced temperature usually helps to control metabolic fluctuations and protein synthesis rate thereby avoiding the formation of insoluble protein aggregates typical of cells at higher growth rate [23]. In the case of CYP153A6 expression, post-induction temperatures of 25°C and above yielded very little or no properly folded active P450. To further improve protein concentration and possibly improve gene expression, a glucose-based defined medium was investigated due to an expected increase in biomass concentration [24]. This necessitated varying inducer concentration (IPTG), as previous investigations have suggested that inducer concentration should be determined on a basis of biomass concentration [25]. The concentration of δ-ALA which is required for heme synthesis was varied simultaneously across the same range. Again, increased concentration of active biocatalyst does not necessarily guarantee the higher hydroxylation rate [19]. Previous studies involving the use of *E*. *coli* as whole cell biocatalyst in alkane hydroxylation have also shown that specific activity was independent of expression levels of alkane mono-oxygenase in different *E*. *coli* hosts [26]. These observations thus support findings from a previous study that microbial physiology may indeed be the overall limiting factor in CYP catalysed bioprocesses [20,21]. Hence, further investigations were aimed at understanding the bioprocess limitations that contribute to cell physiology in octane bio-oxidation.

### Hydroxylation of *n*-octane in a batch and fed-batch reaction using cells cultured on complex LB and glucose-based defined medium

The key bioprocess parameters and results presented in Table 2 shows a relatively improved process for 1-octanol production using resting whole *E*. *coli* cells expressing cytochrome P450 in this present study. The highest biocatalyst activity(16 μmol g_DCW_^-1^ min^-1^) in this study at 37°C presents a minimum of 4-fold increase over data from previous studies using the CYP153 protein in alkane hydrolysis. CYP153A6 is unfortunately not stable at this temperature and the activity was maintained for only 6 h. The addition of glycerol to the biotransformation mixture did not alleviate biocatalyst instability, even though it is often regarded to have a stabilising role in recombinant *E*. *coli* - CYP450 biocatalysis systems [17]. Therefore, further experiments were conducted at 20°C to understand other factors influencing octane hydroxylation activity.

A fed-batch mode of glucose addition was adopted to ease overflow metabolism as by-products such as acetic acid reduce reaction pH and also inhibit metabolism [27,28]. This fed-batch system led to reduced acetic acid formation (which was prevalent in the batch system at glucose concentration above 1 g g_DCW_^-1^), thus improving the coupling of glucose usage to product formation which is essential for enhanced process efficiency [22]. In addition, this reduces the potential formation of octylacetate (condensation of octanol with acetate) which has been observed at very high glucose concentration in a batch system (Smit, personal communication). Further process intensification identified product toxicity as a limiting factor. Using cells grown on LB medium, it was observed that 1-octanol concentrations as low as 0.25 g g_DCW_^-1^ inhibited biocatalyst activity when added at the start of the reaction. However, the cells were able to tolerate increasing 1-octanol produced during the reaction up to 0.8 g g_DCW_^-1^ (Figure 6A.) It suggests the ability of the whole cells to adapt the cell membrane to the toxic nature of the increasing concentration of product secreted into the medium. Solvent toxicity is usually described using the logarithm of the partition coefficient *P* of a particular solvent between a standardised 1:1 mixture of octanol and water, log *P*_*OW*_[29]. Low log *P*_*OW*_ solvents (log *P*_*OW*_ < 4) are regarded as highly toxic due to their relative water solubility and ease partitioning within the cell membrane. This causes membrane fluidity and permeability leading to leakages, disruption in energy metabolism and cell death. Octanol has a log*P*_OW_ of 2.9, hence the need to alleviate its impact on the cell cytoplasmic membrane where it accumulates preferentially. Toxicity of octanol was relieved by the addition of BEHP which acted as a co-solvent and product sink. This prolonged the time that the cells remained catalytically active and increased the final octanol levels obtained with LB grown cells from 0.80 to 1.25 g g_DCW_^-1^ after 60 h. It had an even more pronounced effect on the conversions obtained with cells grown in chemically defined medium. In this case the cells without BEHP remained active for only 48 h, while the cells with BEHP added maintained activity for 120 h, so that a final octanol production of 1.45 g g_DCW_^-1^ was obtained, compared with only 0.65 g g_DCW_^-1^ in the absence of BEHP. The addition of the BEHP did not affect the reaction rate but extended the time over which the cells remained active. Notable was the lower reaction rates obtained with cell grown in chemically defined medium (2.40 μmol_octanol_ g_DCW_^-1^ min^-1^), compared with LB grown cells (4.2 μmol_octanol_ g_DCW_^-1^ min^-1^), even though the CYP153A6 expression was similar (0.20 μmol g_DCW_^-1^). This may be explained by the difference in glucose uptake rate, 0.48 and 0.30 mmol (g_DCW_ h)^-1^ for LB and defined medium cells respectively. It is supported by the similar octanol yield on glucose of 0.49 ± 0.06 mmol^-1^ mmol^-1^ in both systems. Glucose metabolism is directly coupled to cofactor regeneration and cell energy which are required for effective substrate conversion [30]. Other effects might also include the presence of essential metabolites in complex medium which may have aided the cofactor pool or improved flux of NAD(P)H to the enzyme system thus influencing the product formation rate [31]. The physiology conferred on the cells grown in defined medium may also explain the different metabolic pattern shown during octane biotransformation. Although rate of octane hydroxylation was slower to reach its peak, the decrease was also slower, with the cells actively producing octanol up to 120 h, a 2.5-fold increase over duration achieved with LB grown cells. It is likely that this pattern shown in octane hydroxylation may be due to cell metabolism as depicted by glucose uptake rate. The cessation of octanol accumulation using the complex LB and defined medium cultured biomass is apparently due to the decrease in the pH of the reaction mixture from pH 7.2 to 5.4 ± 0.2.

Attempts to improve biocatalyst activity by pre-treating with acetone to permeabilise the cells yielded up to 2-fold increase except where LB grown cells were used at the higher temperature of 37°C. The latter could be due to toxicity arising from high metabolic rate afforded by the medium and temperature, aggravating the cell stress caused by the compromised cell membrane and structure. It has been shown that cells with higher metabolism are more prone to chemical inactivation [32]. This result suggests an apparent limitation of octane mass transfer across the cell membrane. A similar effect of uptake limitation has been reported for biotransformation of octane [33] and *n*-dodecane [34] using *E*. *coli* expressing alkane monooxygenase. Octane hydroxylation rate was improved by 4 fold through cloning an additional protein, AlkL, in the outer membrane of the *E*. *coli* cells to facilitate efficient uptake of the substrate [33]. The authors reported a better result using this system over cell permeabilisation via chemical agents. This shows that further strain design may be necessary for enhanced activity.

## Conclusion

This study provides the first detailed basis for process intensification involving the use of CYP153A6 for the hydroxylation of linear alkanes which is regarded as the physiological substrate of CYP153s. The maximum rate of 1-octanol formation of 16 μmol g_DCW_^-1^ min^-1^ in this system compares favourably with studies using alkane hydroxylase (AlkB) expressed in *E*. *coli*. With the latter, the product is usually subjected to further product oxidation and degradation, negatively affecting downstream processing cost [34]. Hence the CYP153A6 system is favoured. The present study shows that the success of this CYP process is not solely dependent on the concentration of the active monooxygenase, but also on other process conditions with cell physiology playing an important role. Composition of growth medium and supply of nutrients (affecting cofactor pool and regeneration), optimum process conditions (induction and temperature studies) and process engineering (fed-batch supply of glucose and *insitu* product removal) were necessary to enhance biocatalyst efficiency.

Blank *et al*. [35] estimated a maximum specific activity of 370 μmol g_DCW_^-1^ min^-1^ for resting *E*. *coli* cells in oxygenase biotransformation, based on an equimolar NADH/product stoichiometry at a glucose uptake rate of 2.4 mmol (g_DCW_ h)^-1^ allowing maximal NADH yields on glucose. From this prediction, it is apparent that the maximum hydroxylation activity achieved in this study was not limited by the metabolic capacity of the whole cell biocatalyst. This illustrates the ongoing scope for process improvement through biocatalyst and bioprocess design to favour increased CYP153A6 activity and stability.

## Materials and Methods

### Chemicals, bacterial strains and plasmids

Chemicals and antibiotics were obtained from Sigma-Aldrich. *E*. *coli* BL21(DE3) pET28b-PFR1500 containing the CYP153A6 operon from *Mycobacterium sp*. HXN-1500 cloned into plasmid pET28b(+) was used as biocatalyst [19].

### Pre-cultures, medium preparation and expression of CYP153A6

Luria Bertani (LB) broth (tryptone 10 g l^-1^, yeast extract 5 g l^-1^ and NaCl 10 g l^-1^) supplemented with 30 μg ml^-1^ kanamycin was inoculated with *E*. *coli* BL21(DE3) pET28b-PFR1500 glycerol stock (previously maintained at -60°C). The pre-inoculum (50 ml in a 500 ml shake flask) was incubated at 30°C and 160 rpm for 12 h. This was used to inoculate LB medium or defined medium adapted from Pflug et al. [14] containing 10 g l^-1^ glucose at 2% v/v (using a working volume of 200 ml in a 2 L flask). This was supplemented with 30 μg ml^-1^ kanamycin and incubated at 37°C with constant shaking at 160 rpm. At A_578nm_ of 0.6 – 0.8, the medium was supplemented with, unless stated otherwise, 0.5 mM δ-aminolevulinic acid (δ-ALA), 50 μM FeCl_3_.6H_2_O and 0.5 μM isopropyl-β-d-thiogalactopyranoside (IPTG). Cultures were further incubated at 20°C, 160 rpm for a total of 24–26 h after inoculation.

### Studies on CYP153A6 expression and whole cell activity

The effect of inducer concentration (0 – 1.0 mM IPTG) and concentration of heme precursors (FeCl_3_.6H_2_O and δ-ALA, 0 – 1.0 mM) on the expression of CYP153A6 was investigated. These compounds were added at A_578nm_ of 0.6 – 0.8 and cultures were incubated at 20°C. In another set of experiments, the cultivation temperature preceding and following induction was varied from 20 to 37°C.

### Whole cell biotransformation

Following protein expression, the *E*. *coli* BL21(DE3) cells were harvested through centrifugation at 7000 *g* and 4°C for 10 min. The cell pellet was resuspended in 200 mM sodium phosphate buffer (pH 7.2) containing glucose and 100 μg ml^-1^ FeSO_4_.7H_2_O. This biotransformation reaction mixture (BRM) was used for octane biotransformation under non-growing, but metabolically active conditions. One millilitre aliquots of this mixture was transferred into 40 or 60 ml sterile amber screw cap vials and 300 μl of *n*-octane added to each vial before capping and placing on an orbital shaker at 200 rpm and 20°C (unless otherwise stated). Vials were removed at specified time intervals for product extraction. The biotransformation reactions were stopped by adding 100 μl of 5 M HCl to each vial. The octanol, residual glucose and acetic acid concentrations were determined.

### Influence of bioprocess conditions on biocatalyst efficiency

Non-growing, but metabolically active cells were used in studying the influence of various bioprocess parameters such as temperature, overflow metabolism, product toxicity and biocatalyst stability on octane biotransformation. The glucose concentration at the start of reaction was varied from 0.5 to 2.5 g_glucose_ g_DCW_^-1^. The preferred concentration was used in another set of experiments to study the influence of reaction temperature (20 - 37°C) on product accumulation and CYP stability. For the toxicity studies, a carrier solvent into which the product was selectively drawn was added using hexadecene or bis(2-ethylhexyl) phthalate (BEHP), due to properties such as recovery efficiency, non-toxicity to *E*. *coli* cells and no interference with reaction mixture [36]. To 1 ml of re-suspended biomass, 100 μl of the carrier solvent and 200 μl of octane was identified as the preferred ratio and added to the reaction mixture.

### Effect of cell permeabilisation on whole cell biocatalyst activity

To determine the extent of mass transfer in the reaction vials, studies were carried out by pre-treating (permeabilising) the whole cell biocatalyst with acetone and toluene in the range of 5 to 20% (v/v). The permeabilising agents were added to the re-suspended cells (in 200 mM sodium phosphate buffer, pH 7.2) and vortexed in an Eppendorf tube for 2 – 3 mins. A 1 ml aliquot of the pre-treated cells was transferred into the 60 ml reaction vial containing 300 μl of octane and 40 mM glucose (final concentration). This was mixed on an orbital shaker at 200 rpm while temperature was maintained at either 23 or 37°C.

### Analytical Methods

#### Quantification of cell growth and substrate utilisation

Biomass concentration was measured gravimetrically as cell dry weight and by absorbance at A_578nm_. Glucose utilisation was analysed by determining the generation of H_2_O_2_ spectrophotometrically, following reaction catalysed by glucose oxidase using Roche glucose oxidase kit.

#### Quantification of whole-cell CYP153A6

The functional CYP153A6 protein in whole cells was quantified using CO-difference spectra [37]. Cell suspension samples of 1000 μl were placed in 10 ml test tubes and saturated with carbon monoxide. The samples were reduced immediately by adding sodium dithionite powder. The absorbance spectra between 400 and 500 nm of reduced samples with and without CO were recorded using a UV-Vis spectrophotometer (UNICAM Helios α). The A_450-490_ difference was determined and the active concentration of the CYP153A6 protein was calculated using an extinction coefficient of 91 mM^-1^ cm^-1^.

#### Analysis of overflow metabolism through acetic acid accumulation

The concentration of acetic acid was quantified by high pressure liquid chromatography (HPLC) using Aminex HPX-87H column (Bio-Rad). The operating temperature was 60°C. Separation was achieved using 0.01 M H_2_SO_4_ at a flow-rate of 0.6 ml min^-1^ and analysed by UV absorbance at 210 nm.

#### Analysis of octanol formation

The contents of each vial were extracted using 1000 μl of ethyl acetate containing 0.3% 1-decanol (v/v) as the internal standard. GC analysis was carried out using a Varian 3900 series gas chromatograph equipped with a flame ionisation detector and a non-polar Varian factor four column (VF–1 ms) composing of dimethylpolysiloxane, measuring 15 m × 0.25 mm. The inlet temperature was 280°C, the flow of nitrogen (carrier gas) through the column was 15 ml min^-1^ and the sample volume was 1 μl. The temperature program used was adapted from Gudiminchi *et al*. [19]: isotherm maintained at 120°C for 5 min, temperature ramped at 20°C min^-1^ to 280°C, followed by a 7 min isotherm. Product concentrations were calculated using a standard curve of 1-octanol in the range 5 to 100 mM.

## Competing interests

The authors declare that they have no competing interests.

## Authors’ contributions

OAO did the experimental design, carried out all the experiments and drafted the manuscripts. CJF participated in experiment design and drafting of the manuscript. RKG participated in experiment design. MSS and STLH conceived of the study, and participated in its design, coordination and drafting of the manuscript. All authors read and approved of the manuscript.
